# Mitigation Policy Acceptance Model: An Analysis of Individual Decision Making Process toward Residential Seismic Strengthening

**DOI:** 10.3390/ijerph15091883

**Published:** 2018-08-30

**Authors:** Hao-Teng Cheng, Ko-Wan Tsou

**Affiliations:** Department of Urban Planning, National Cheng Kung University, Tainan City 70101, Taiwan; uptkw@mail.ncku.edu.tw

**Keywords:** seismic risk mitigation, affective, mitigation policy acceptance model, behavioral intention, structural equation modeling

## Abstract

Mitigation policy is regarded as an effective strategy to achieve the purpose of building health resilience and reducing disaster risk with the current high frequency of environmental event occurrences. To enhance public acceptance of mitigation policy, the issue of decision-making behavior has been a concern of researchers and planners. In the past literature, qualitative measures employed to reveal the behavioral intention of hazard risk mitigation cause restricted outcomes due to the problem of sample representativeness and the fact that quantitative research is restricted to discuss the linear relationship between the two selected variables. The purpose of this article is to attempt to construct a Mitigation Policy Acceptance Model (MPAM) to analyze the behavioral intention of seismic risk mitigation strategies. Based on Dual Processing Theory, affective is conducted as the core variable for constructing two types of thinking processes, and the variables of risk perception, trust and responsibility are selected in MPAM from theories and past research. In this study, the mitigation policy of residential seismic strengthening, adapted in Yongkang District of Tainan, has been conducted as the case study. According to the results, the result of model fit test has confirmed the MPAM framework, and two thinking modes could be associated together when people face a risky decision-making process. The variable of affective is the most effective factor to influence each variable, and a direct effect on intention is also shown in this model. The results could provide suggestions in communication risk strategies for the government.

## 1. Introduction

Building city resilience has been a concern of governments in order to increase the capacity of spatial health systems through risk communication to reduce the negative impacts of shock on public health [1]. The Sendai Framework for Disaster Risk Reduction 2015–2030 outlines the strong relationship between health and disasters with the high frequency of unpredictable natural hazards in recent years [2,3]. The achievement of enhancing city resilience through disaster risk reduction is seen as an effective method for decreasing the possible damages [4] to public health and integrating the spatial health systems in order to reduce the pressure when disasters occur. Overall, many studies have been devoted to this topic through various views, such as developing new technical methods to predict the possibility of hazard occurrence or providing a new framework to measure different types of vulnerability [5,6,7], for advancing the concepts of risk by a new paradigm shift or methodology breakthrough [8,9,10]. In recent years, substantial attention in public action towards environmental mitigation policies, resulting from the serious threat to public health, has been observed by scholars. From the perspective of public health, the public realizing how to respond to natural hazards is important in order to build city resilience [1,4,11,12] and achieve successful policy implementation. Residential Seismic Strengthening seems to be an effective mitigation policy worldwide for adoption to reduce the disaster losses by building collapse [13]. The importance of seismic retrofitting could be conducted by past research [13,14].

From the perspective of the subjectively expected utility model in Financial Decision Theory, people might be willing to support public policies for reducing the threat if decision-makers adhere to axioms of rationality. In other words, people could regard adoption behavior as an investment target and be willing to support it with considerations towards the cost and benefit. Some famous theoretical models such as the Health Belief Model (HBM) or Protection Motivation Theory (PMT) are grounded on a perspective of consequentialism for seeking the greatest effectiveness. In the process of rational consideration, the assumption is based on the complete information provided to make individual decisions. However, in general, the threat of environmental events could not be predicted completely. Recent natural hazards, such as earthquakes or hurricanes around the world, are consistent with the unpredicted threats from natural events. The uncertainty information may lead to susceptibility to the threat. As a result, people could rely on prior experience or life experience by making decisions through different considerations rather than a rational consideration process [15,16]. 

With the breakthrough of technological measurements, psychologists have confirmed a different type of consideration to be able to answer this problem of how to explain a risk decision-making process. Evans (2003) [17] combines relative research results and presents Dual Processing Theory (DPT) to describe two kinds of consideration systems that could exist at the same time. Furthermore, Slovic et al. (2004) [18] describes two separate active systems when facing risky decision making. One is to realize the risk by logistic and rational analyses, and the other is to realize the risk by intuitive feeling and quick-thinking. In these two modes, the influence of the affective is shown by serial relative theories through psychological experiments within the process of decision making. In past research, affective is a measurement of variables (rather than a variable) to discuss the relationship with seismic mitigation adoption behavior. Most past studies apply affective to measure the risk perception of seismic hazards instead of an independent variable [19,20,21,22,23]. Seldom results are found in discussing the structural relationships between affective and adoption behavior. The past rational behavior-based theories such as Theory of Planned Behavior (TPB) or PMT have also had restrictions applied to them when explaining risk decision-making.

The successful implementation of environmental mitigation policy relies on strong public support. Research has been conducted to confirm the factors affecting public intention to adopt seismic mitigation policies by quantitative analysis, such as correlation analysis [24,25], multiple regression [26], and logistic regression [22]. Additionally, parts of research, through interview-based methods, reveal the decision-making framework for discussing the relationship between each affecting factor [27,28,29]. Past research has shown the importance of seismic risk management in environmental planning [30] and it provides the foundations for a deeper discussion in determining the structural relationships of each decisive factor.

The aim of this study is to construct a new model, named Mitigation Policy Acceptance Model (MPAM), for analyzing the behavioral intention of mitigation policy acceptance. The theoretical framework of MPAM is based on the Dual Processing Theory. The variable affective is the critical driving factor for connecting the pathways and variables such as risk perception, trust and responsibility from past individual decision-making theories and literature are also selected in this model. The structural equation modeling (SEM) is employed as the methodology for (1) testing the availability of MPAM, (2) realizing the thinking process of decision making under risk, and (3) analyzing the structural relationship of each selected variable. The residential seismic strengthening strategy adopted in Yongkang District of Taiwan is conducted as the case study of seismic mitigation policy through questionnaire surveys. This article includes five parts: Section 1 is the Introduction. Section 2 introduces the case study, MPAM framework, and research hypothesis. Section 3 explains the results of MPAM and Section 4 discusses these results. The final section makes some conclusions.

## 2. Materials and Method

### 2.1. Framework of MPAM 

**Figure 1:**
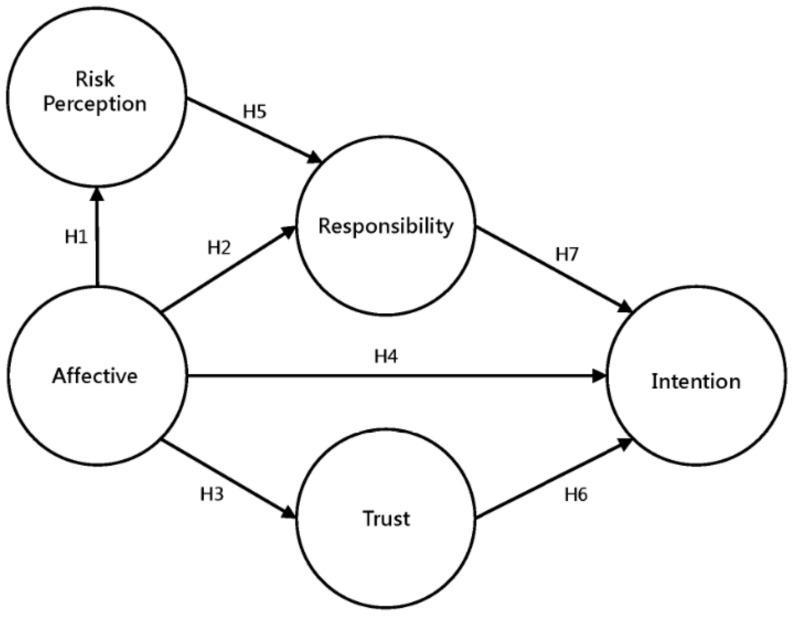
The Framework of MPAM.

The framework of the new model in this study is based on the Dual Processing Theory for connecting the pathways of each latent variable in SEM. Three main concepts are shown in the framework of MPAM: (1) The variables, including risk perception, affective, and trust, are selected by past theories and the literature of policy acceptance and seismic preparedness to reveal the character of risky decision making with the intention of policy acceptance mitigation; (2) the variable affective is regarded as the core factor to connect each thinking process. In the rational thinking mode, affective may be regarded as the information required to change individual perception and attitude and has an indirect impact on behavioral intention. In the automatic thinking mode, affective may be the kind of feelings required to drive personal decisions and has a direct impact on behavioral intention; (3) structural relationship of pathways with two types of thinking processes, including rational thinking and automatic thinking, are formed by individual decision-making theories and psychological hypotheses. The former includes the Person Relative to Event (PrE) and the Affect-as-Information Theory. The latter contains the “Affect Heuristic” and “Risk as Feelings” hypotheses. The research hypotheses are outlined as follows and are represented in Figure 1.

**Hypothesis** **1 (H1).** *The affective of seismic hazards may influence individual risk perception.*

**Hypothesis** **2 (H2).** *The affective of seismic hazards may influence the responsibility of seismic mitigation adoption.*

**Hypothesis** **3 (H3).** *The affective of seismic hazards may influence the trust of government information and policies.*

**Hypothesis** **4 (H4).** *The affective of seismic hazards may influence the acceptance intention for seismic mitigation policies.*

**Hypothesis** **5 (H5).** *The risk perception of seismic hazards may influence the responsibility of seismic mitigation adaption.*

**Hypothesis** **6 (H6).** *The trust of government information and policy may influence the acceptation intention for seismic mitigation policies.*

**Hypothesis** **7 (H7).** *The responsibility of seismic mitigation adaption may influence the acceptation intention for seismic mitigation policies.*

### 2.2. Variable Measurement and Data Resource

To confirm the new model presented in this study, the behavioral intention of seismic mitigation policy acceptance of the survey area residents were realized through the questionnaire. The survey was conducted between 23 October and 20 December 2014. There were a total of 417 effective survey responses, and the effective response rate was 92.7%. The respondents to the survey were reminded of some notifications. Seismic hazards are defined as an over 6.0 magnitude earthquake. The measurement of each variable is listed in Table 1. Each variable is measured by using at least three questions or more as Table 2. The behavioral intention of adopting residential seismic strengthening policies is the dependent variable. Independent variables—risk perception, affective, trust, and responsibility—are selected by considering the characteristic of adopting seismic mitigation policies and paying high costs. The descriptive statistics for each variable are shown in Table 3. The introduction of each variable is explained as follows.

#### 2.2.1. Risk Perception

Risk perception is the recognized perceived loss in a specific environment [48] and the risk perception of natural hazards is defined as peoples expectation of the probability of having negative outcomes (such as death, injury, property loss, and life disturb) from extreme environmental impacts. In other words, individual risk perception is framed by the expectation of whether or not hazards may happen and cause losses [49,50]. Risk perception is employed as an essential factor in explaining earthquake preparedness behavior in numerous studies [24,25,42,47,48]. 

In fact, the variable of risk perception has been measured with various definitions. According to the classification by Pan in 2012 [22], five kinds of meanings could be found in past studies, including the controllability of disaster losses, the visibility of hazard, the fearfulness of hazard, the possibility of hazard occurrence and the severity of disaster losses. However, a compound measurement has been determined by past research and results in diverse outcomes in the effects of behavioral intention or other variables. To identify the outcomes of MPAM, this study measures risk perception by the expectation of the probability of occurrence and having negative outcomes.

#### 2.2.2. Affective

The influence of affective has not been given attention when analyzing behavioral decisions in past research. In recent years, several research studies discussing public risk perception pointed out the relationship between affective and decision behavior [40]. Joffe et al. (2013) [51] emphasized the effect of affective on risky decision processes. The affective of natural hazards could raise a person’s risk perception to support mitigation adoption. This influence is also confirmed by theories of psychology, such as Dual Processing Theory [18]. Affective seems to be a feeling of hazards in the risk decision process.

#### 2.2.3. Trust

Trust is a personal feature that could arouse positive expectation in a relative position [52,53]. James (2000) [54] considers trust as a risky benefit of trade that trust agents could not have in Speculative behavior. The definition of trust in public policy implementation may include the accuracy of official information and the implementation of policy. Paton et al. (2010) [55] determines that the trust of political, scientific prediction, and morality may promote the public to adopt seismic adjustments. Several theories, such as the Health Belief Model and Protection Motivation Theory, provide significance to trust when discussing risky decision behaviors.

#### 2.2.4. Responsibility 

Past research has confirmed the effect of responsibility on hazard adjustment adoption [35,36]. The responsibility in hazard adjustment adoption is an important concept to let people make decisions. People could think of whether the adoption behavior is responsible for them when facing threatening natural hazards. The uncontrollable characteristics of natural hazards could change the view of accepting mitigation policies resulting from the doubt of whether people should pay money by themselves or from governments [56]. Responsibility is also regarded as an important factor in PrE theory.

In sum, the framework of MPAM has some advantages to analyze the decision-making process of environmental mitigation policies: (1) Two kinds of thinking processes constructing the whole model respond the characteristic of risky decision making under information uncertainty; (2) the selected variables reveal the topic of mitigation policies acceptance; (3) the hypnosis of the causal path is based on theoretical foundation from interdisciplinary research and theories to ensure the reliability and availability modeling; (4) affective is the key factor to observe how to make the decision through two thinking systems. These traits could be determined by SEM and are reexamined in the Discussion.

### 2.3. Case Study Area 

Taiwan is located at the Circum-Pacific seismic zone. According to official statistics, there were 3197 over 4.0 magnitude quakes that occurred in Taiwan Island in the past two decades. The official Seismic institution has predicted that there is a high possibility of a large earthquake happening in the future in Yongkang City. An over 5.0 magnitude quake occurred in February of 2016, bringing hundreds of casualties resulting from building collapses. According to the official statistics, approximately 10 thousand building cannot bear a high magnitude quake because of their low seismic capabilities. In this context, the governments, through providing subsidies, attempted to encourage individual seismic house strengthening.

## 3. Results

The results of the model fit test are shown in Table 4. Five common statistic tests of SEM are applied in this study, including χ^2^, χ^2^/DF, GFI, AGFI and RMSEA. All the statistic tests, besides the *p*-value, passed, and it seems to show that the framework of this model has a good fit. In general, the value of χ^2^ may change obviously when the sample number is large. The value of RMSEA is lower than 0.05, and shows the great fitness of MPAM. In other words, the structural framework of MPAM is conducted by the results of the statistic tests. Table 5 shows the results of the path analysis by SEM. The causal paths in the framework of MPAM have been explained in Table 1. The effects of affective on risk perception, trust, and responsibility have the statistical significant. All the impacts of all the factors on intention do not pass the statistical test; however, these results do not mean that there would have been no relationships between each variable and intention if the test of model fit had passed. There are two possible reasons to explain this result. The first is that a nonsignificant relationship could change in responders from other case study areas. The second is that the relationship could be present in specific groups of responders. The full results of the hypotheses are shown in Table 6. The H1, H2, H3, H4, H6, and H7 are confirmed according to the test of model fit. The H5 hypothesis does not shown the necessity when determining the best model framework in the principle of the model selecting process.

In the effect analysis of SEM (Table 7), the factor of affective has deep effects on each variable according to the result of MPAM. The highest effect on risk perception shows a close relationship, and a negative influence on responsibility is shown in this model. A direct effect of affective, trust, and responsibility on intention is shown in this model.

According to the results of SEM, the MPAM based on the Dual Processing Theory concludes some findings. Firstly, two different thinking modes truly exist when facing a risk decision-making process. In the case of residential seismic strengthening strategies, people would analyze the whole situation and conditions when they need to spend lots of time and money. On the other hand, people may also determine the decision by the affect heuristic mode when facing a decision between risk and uncertainty. This confirms that a rational-behavior-based theory is not enough to explain the whole public decision-making process. Secondly, the selected variables of trust and responsibility have an impact on behavioral intention. These results confirm the importance of these two variables in the past literature, of whether people are willing to accept public policies with uncertainty or not. Thirdly, the variable of affective has direct and indirect effects on behavioral intention, revealing the fact that the importance of this core variable has a significant impact on both rational and automatic thinking modes.

## 4. Discussion

This study attempted to present a new model to realize the intention behavior when people faced risky decision-making. According to past psychological research, affective has been confirmed to have an influence on intention behavior [19,31,36]. To classify the influence of affective in the decision-making process, the model hypothesis based on past psychological theories and seismic preparedness behavior research focused on the influence of affective. 

According to the results of SEM, some discussion about the relationships may be shown in the following context. From the results, Hypothesis 2 has a negative effect on the factor of responsibility. A different result which is inconsistent with the preview hypothesis is shown in this study [41,43]. According to past research, the public attitude of the responsibility for adopting hazard mitigation policies might be based on the characteristic of natural hazards if natural hazards are so threatening that people could not handle the possible damages when earthquakes happened. In other words, heavy negative affective could let people change their way of thinking to do something on their own. In addition, the positive effect of affective on intention is shown in this study, a different result from past research. Positive and negative effects are all shown in the past research. Rüstemli and Karanci (1999) [41] and Heller et al. (2005) [43] confirm the positive result and Basolo et al. (2009) [27] shows a negative result. The reason for explaining the inverse outcomes is fatalism [42]. The cultural difference between natural hazards could have a critical impact on behavior [57]. As a result, fatalism explains why people could do nothing instead of adopting seismic mitigation policies. Finally, Hypothesis 5 does not pass the statistical test of the model fit. This result could be explained through the effect of responsibility on behavioral intention. When considering seismic mitigation as responsible for governments, people may not spend extra money to reduce the damage from hazards before they happen. As a result, risk perception does not have a significant impact on the responsibility. 

MAPM confirms two thinking process exist when people make decisions on environmental mitigation policies with uncertain information. People may have an intuitive feeling or rational thinking that affects their behavioral intention. Also, the pass of model fitness shows the reliability and availability of variable selections and causal structure in this model framework. In the past literature, many researchers have devoted themselves to conducting the effects of policy incentives or personal social characteristics on mitigation policy acceptances for enhancing the effectiveness of implementing policies. In fact, people may support public policies through quick-thinking rather than logistic and rational thinking process. The results are shown for the direct and indirect impacts of affective on the behavioral intention in the study area. In this case, governments should think how to release public anxiety and worries of uncertainty through effective risk communication for achieving a successful implementation and adaption. In sum, the framework of MAPM provides a different way to analyze public willingness of environmental mitigation policies, and it is helpful for governments to develop risk communication strategies.

## 5. Conclusions

The study on exploring the process of whether people are determined to adopt mitigation policies has been a concern of governments with the high frequency of environmental hazards occurring in recent years. The re-emphasis is on mitigation, and preparedness in modern disaster risk-management arouses more importance in the characteristics of the dramatic consequences brought on by extremely hazardous events. In this study, MPAM-based Dual Processing Theory is proposed to reveal the structural interaction between the selected factors and mitigation policy acceptance to explain the thinking process of the public from automatic or rational systems as a response to seismic hazards. A case study area of Yongkang City in Taiwan is conducted to analyze the decision making process of residential seismic strengthening policy. The great model fitness (RMSEA < 0.05) is shown by the statistical tests, and the effect of affective is emphasized in the model of this article for explaining the structural decision process and has been confirmed by the results in this paper. The affective has a direct impact (0.221) and an indirect effect (0.005) on the intention. 

On the whole, MPAM shows the availability to analyze the decision-making process and confirms that people may support public policy through quick-thinking process rather than logistic consideration when facing uncertainty. The question why the past rational-based model could not completely explain the behavior of supporting public policies could be answered, even if it is helpful for people to reduce the losses and decrease the reduce the risk potential to life. In this study, a preliminary result of MPAM is shown for analyzing the individual decision-making process of residential seismic strengthening policy. The future work could apply MPAM in comparison with an existing theoretical model through different study areas or mitigation policies for realizing the potentiality and restriction of the new model. The results in the paper could suggest to the government how to make risk communication strategies for residents. It is believed that it could be helpful for enhancing health resilience and reducing the pressure of spatial health systems through reducing negative impacts of future environmental hazards.

## Figures and Tables

**Table 1:** The set of pathways, expected symbols, theories, and literature.

Hypothesis				Expected Symbols	Theory or Hypothesis	Past Literature
H1	Affective	→	Risk perception	+	Affect Heuristic [31]	[32,33,34,35]
H2	Affective	→	Responsibility	+	Affect-as-information theory [36]	[37]
H3	Affective	→	Trust	+	Affect-as-information theory [36]	[38,39]
H4	Affective	→	Intention	+	Risk as feelings [39]	[22,26,29,40,41,42,43]
H5	Risk perception	→	Responsibility	+	PrE [44]	[45]
H6	Trust	→	Intention	+	-	[26,41,46,47,48]
H7	Responsibility	→	Intention	+	PrE[44]	[27,28,29,48]

**Table 2:** The variables of the model in this study.

Dependent Variable		
Intention (Yes/No question)		
Y1	Y1_1	Willing to take an initiative to adopt residential seismic strengthening.
	Y2_2	Willing to adopt residential seismic strengthening policies with subsidies.
	Y3_3	Willing to adopt residential seismic strengthening policies with subsidies when the building’s seismic capability is deemed to be not enough after an official evaluation.
**Dependent Variables**		
Risk Perception (Yes/No question for 1, 3, and 5; 5 point Likert Scale of 2 and 4).		
X1	X1_1	Perceived Probability to experience an over 6.0 magnitude earthquake in 10 years.
	X1_2	The certainty of the answer for the Perceived Probability.
	X1_3	Perceived Loss from experiencing an over 6.0 magnitude earthquake in 10 years.
	X1_4	The certainty of the answer for Perceived Loss.
	X1_5	A past experience of loss by a natural hazard.
Affective (5 point Likert Scale)		
X2	X2_1	Fear of an earthquake.
	X2_2	Worry of an over 6.0 magnitude earthquake occurring in 10 years.
	X2_3	The fear of building collapse when an over 6.0 magnitude earthquake happens.
Trust (5 point Likert Scale)		
X3	X3_1	The trust in the seismic information by governments.
	X3_2	The trust in the seismic warnings and predictions by experts.
	X3_3	The trust in adopting hazard mitigation policies by governments.
Responsibility (5 point Likert Scale)		
X4	X4_1	The option of providing subsidies when people lose items due to earthquakes.
	X4_2	The option of providing subsidies when people lose items due to earthquakes with an official warning.
	X4_3	The option of the responsibility for paying an extra fee when governments adopt seismic strengthening policies with subsidies.

**Table 3:** The descriptive statistics for each variable.

Variable Name	Minimum	Maximum	Mean	Std. Deviation
X1_1	0	1	0.53	0.500
X1_2	1	7	4.00	1.042
X1_3	0	1	0.82	0.385
X1_4	1	7	4.60	1.186
X1_5	0	1	0.34	0.473
X2_1	2	7	4.93	1.227
X2_2	1	7	4.58	1.212
X2_3	1	7	4.58	1.287
X3_1	1	7	4.19	0.930
X3_2	1	7	4.42	0.852
X3_3	1	7	4.17	1.058
X4_1	1	7	2.36	1.071
X4_2	1	7	2.92	1.186
X4_3	1	7	4.61	1.148
Y1_1	0	1	0.45	0.498
Y1_2	0	1	0.83	0.375
Y1_3	0	1	0.93	0.251
Gender	0	1	0.44	0.497
Age	1	6	3.74	1.572
Education	1	6	3.49	1.402
Income	1	4	1.50	0.728
House age	1	6	4.01	1.534

**Table 4:** The test of model fit.

	Standard Fit	Value	Result
χ^2^	*p* > 0.05	95.822 (*p* = 0.00)	fail
χ^2^/DF	<3	1.597	pass
GFI	>0.9	0.966	pass
AGFI	>0.9	0.948	pass
RMSEA	<0.08	0.038	pass

**Table 5:** The result of regression weights.

Causal Path			Estimate	Standardized Estimate	S. E.	C. R.	*p*-Value
affective	→	risk perception	0.074	0.540	0.028	2.685	0.007 ***
affective	→	trust	0.081	0.185	0.031	2.595	0.009 ***
affective	→	responsibility	−0.283	−0.226	0.068	−4.184	0.000 ***
affective	→	intention	0.077	0.221	0.050	1.540	0.124
trust	→	intention	0.150	0.189	0.104	1.440	0.150
responsibility	→	intention	0.037	0.132	0.024	1.535	0.125

*** significant at 0.01

**Table 6:** The result of the hypotheses.

Hypothesis				Result
H1	Affective	→	Risk perception	PASS
H2	Affective	→	Responsibility	PASS
H3	Affective	→	Trust	PASS
H4	Affective	→	Intention	PASS
H5	Risk perception	→	Responsibility	FAIL
H6	Trust	→	Intention	PASS
H7	Responsibility	→	Intention	PASS

**Table 7:** The direct, indirect, and total effects of MPAM.

Variable Name		Effects on				
		Risk Perception	Affective	Trust	Responsibility	Intention
Risk perception	Direct	-	0	0	0	0
	Indirect	-	0	0	0	0
	Total	-	0	0	0	0
Affective	Direct	0.540	-	0.185	−0.226	0.221
	Indirect	0	-	0	0	0.005
	Total	0.540	-	0.185	−0.226	0.227
Trust	Direct	0	0	0	0	0.189
	Indirect	0	0	0	0	0
	Total	0	0	0	0	0.189
Responsibility	Direct	0	0	0	-	0.132
	Indirect	0	0	0	-	0
	Total	0	0	0	-	0.132
Intention	Direct	0	0	0	0	-
	Indirect	0	0	0	0	-
	Total	0	0	0	0	-
